# Evidence for pathogenicity of variant *ATM* Val1729Leu in a family with ataxia telangiectasia

**DOI:** 10.1007/s10048-021-00639-4

**Published:** 2021-03-29

**Authors:** Ali S. Shalash, Thomas W. Rösler, Mohamed Salama, Manuela Pendziwiat, Stefanie H. Müller, Franziska Hopfner, Günter U. Höglinger, Gregor Kuhlenbäumer

**Affiliations:** 1 Department of Neurology, Faculty of Medicine, Ain Shams University, Cairo, Egypt; 2 Department of Neurology, School of Medicine, Technical University of Munich, Munich, Germany; 3 Department of Translational Neurodegeneration, German Center for Neurodegenerative Diseases (DZNE), Munich, Germany; 4 Institute of Global Health and Human Ecology, American University in Cairo (AUC), Cairo, Egypt; 5 Faculty of Medicine, Mansoura University, Mansoura, Egypt; 6 Institute of Clinical Molecular Biology, University of Kiel, Kiel, Germany; 7 Department of Neuropediatrics, University Medical Center Schleswig-Holstein, University of Kiel, Kiel, Germany; 8 Institute of Health Informatics, UCL, London, UK; 9 Department of Neurology, Hannover Medical School, Hannover, Germany; 10 Department of Neurology, University of Kiel, Kiel, Germany

**Keywords:** Ataxia telangiectasia, ATM serine/threonine kinase, Mutation, Pathogenicity, Egypt

## Abstract

Ataxia telangiectasia is a rare autosomal recessive multisystem disorder caused by mutations in the gene of ATM serine/threonine kinase. It is characterized by neurodegeneration, leading to severe ataxia, immunodeficiency, increased cancer susceptibility, and telangiectasia. Here, we discovered a co-segregation of two *ATM* gene variants with ataxia telangiectasia in an Egyptian family. While one of these variants (NM_000051.4(ATM_i001):p.(Val128*)) has previously been reported as pathogenic, the other one (NM_000051.4(ATM_i001):p.(Val1729Leu)) is regarded as a variant of uncertain significance. Our findings in this family provide additional evidence for causality of the second variant and argue that its status should be changed to pathogenic.

## Introduction

The rare multisystem disorder ataxia telangiectasia (AT) usually starts in childhood and causes neurodegeneration leading to ataxia, movement disorders, and peripheral neuropathy [1]. Additional characteristics are variable amounts of immunodeficiency, increased susceptibility to cancer, especially of lymphoid origin, telangiectasia, and several additional symptoms [2]. AT is an autosomal recessive disorder known to be caused by variants in the ATM serine/threonine kinase gene (*ATM*) [3].

In the present study, we analyzed an Egyptian family with AT. The family consisted of non-consanguineous parents and four siblings; three of whom are affected by AT. The brother and youngest sister had presented with ataxia, while another sister had a history of ataxia and died undiagnosed at the age of 6 years. We identified compound heterozygous, most likely causative variants in the *ATM* gene.

## Material and methods

### Clinical phenotyping

Ethical approval was obtained by Mansoura University, Egypt (RP/42), and Technical University of Munich, Germany (203/15s). Written informed consent was obtained for all participants. If the participant was minor or incapable, consent was given by the legal custodian. Neurologists specialized in movement disorders (A.S.S. and G.U.H.) examined affected and unaffected individuals at the Department of Neurology, Ain Shams University, Cairo, Egypt. Affected individuals II:1 and II:4 were examined by standard cranial MRI at 1.5 Tesla with T1, T2, and FLAIR sequences.

### Genetic analysis

Exome sequencing was performed in the two living, affected individuals II:1 and II:4. Genomic DNA libraries were captured using the Nextera Rapid Capture Expanded Exome Kit (Illumina, San Diego, CA), and DNA fragments were sequenced on an Illumina HiSeq2000 system with an average coverage of 80×. Variants were identified by a standard analysis pipeline and annotated using the ANNOVAR software [4]. Since the parents are not known to be related, we assumed autosomal recessive inheritance and compound heterozygosity or homozygosity of the causative variant(s). We discarded the sex chromosomes and variants with a minor allele frequency (MAF) > 0.01 in gnomAD (Genome Aggregation Database) “all” (https://gnomad.broadinstitute.org) exome as well as genome data. We discarded variants without an annotated exonic or splicing function and variants with a CADD score below 15 [5]. Next, we filtered for variants present in both exomes in the hetero- or homozygous state in the genes with the HUGO (Human Genome Organization) official symbols *ATM*, *MRE1*, *APTX*, *SETX*, and *PNKP* implicated in the pathogenesis of AT and its differential diagnoses [2]. This resulted in two variants in the *ATM* gene which were confirmed and tested for segregation in the whole family by Sanger sequencing. Primer sequences are available on request. We detected no variants fulfilling the abovementioned criteria in any of the 4 other genes. The effects of amino acid substitutions on protein function were predicted using MutationTaster [6], PolyPhen-2 [7], and CADD. We searched the public version of the Human Gene Mutation Database [8], ClinVAr [9], and the Leiden ATM mutation database [10] for the identified variants.

## Results

### Case reports

**Fig. 1:**
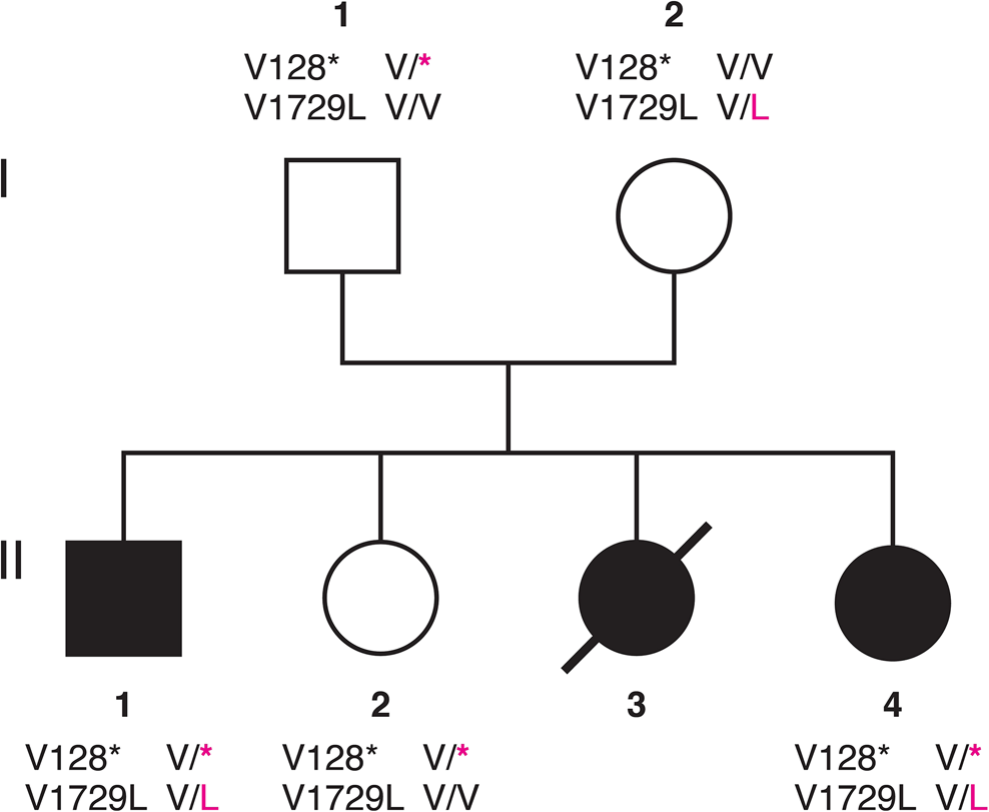
Pedigree of the family with genotypes of variants 1 and 2. Square, male; circle, female; no filling, not affected; black filling, affected; slash, deceased. Exact HGVS nomenclature of variant 1 (V128*) and variant2 (V1729L) is shown in Table 2

**Fig. 2:**
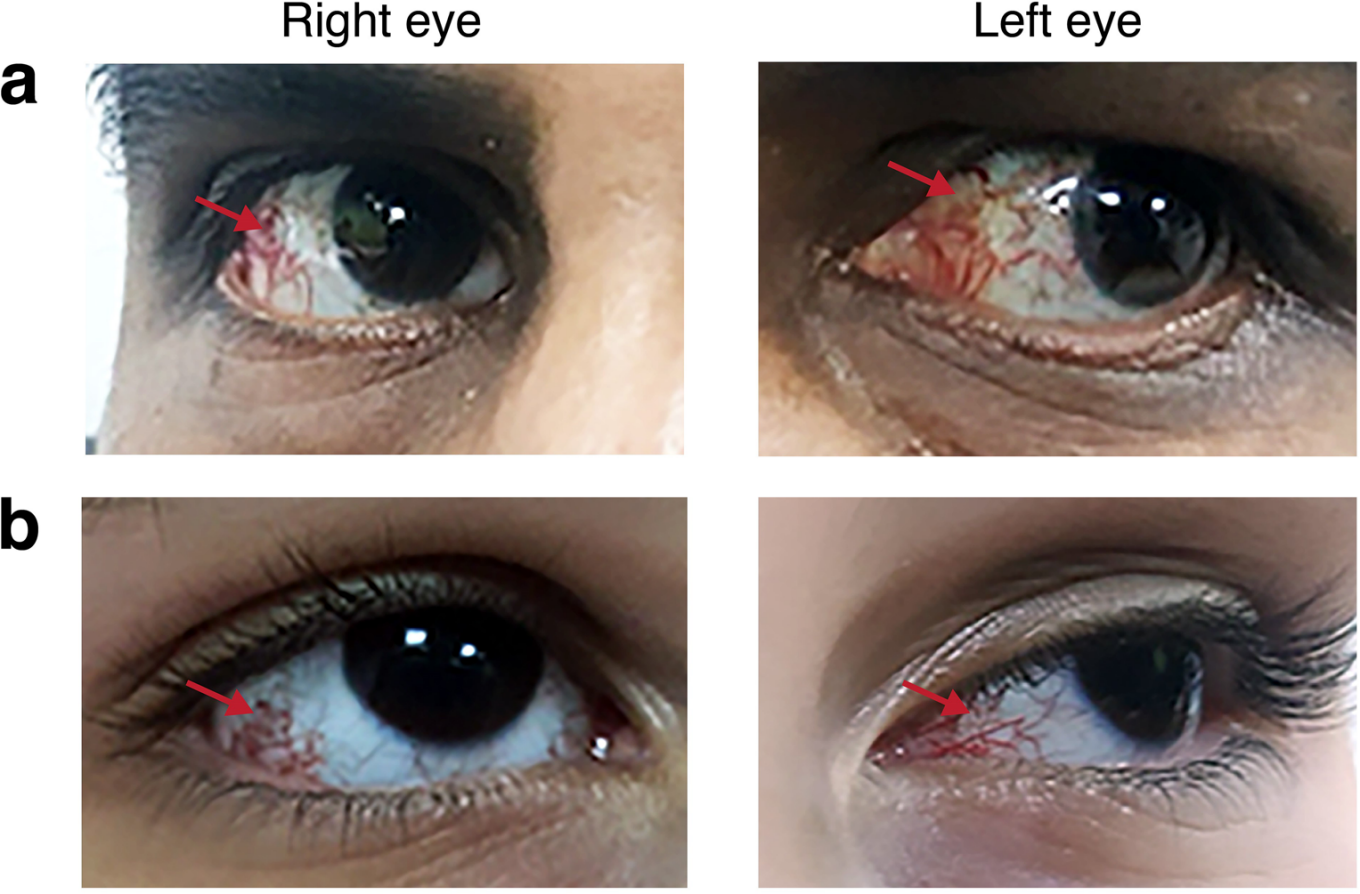
Ocular telangiectasia. Images show prominent blood vessels over the sclera, labeled by red arrow in **a** patient #1 (II.1) and **b** patient #2 (II.4) of the Egyptian family

The detailed clinical findings of both living patients are presented in Table 1, and the pedigree is shown in Fig. 1. Patient 1 (II:1) presented to us at age 23 with gait and limb ataxia, spastic paraparesis with pyramidal signs in the presence of length-dependent peripheral neuropathy. He showed dystonia of the upper limbs as well as the neck, severe dysarthria, oculomotor abnormalities, and cognitive impairment. At age 14, he developed focal seizures. Patient 2 (II:4) is a sister of patient 1 and presented to us at age 11 with a very similar clinical picture but with less prominent dystonia and without epileptic seizures. Both patients showed cerebellar atrophy on cranial MRI. Both patients suffered from recurrent infections and showed conjunctival telangiectasia (Fig. 2a, b) as well as hypopigmented skin patches. Progressive ataxic gait noticed at the age of around 1.5 years was the first symptom in both siblings. Increased levels of alpha-fetoprotein (AFP) were found in both affected siblings. Another sister (II:3) died at age 6 with a similar clinical picture. In addition, she suffered reportedly from hemolytic anemia and was treated with blood transfusions and steroids. The third sister (II:2) and the parents were neurologically healthy.

**Table 1:** Clinical features of the AT patients

Demographic/clinical features	Patient #1 (II:1)	Patient #2 (II:4)
Age at onset (y)	1.5	1.6
Age at first examination (y)	23	11
Age at last follow-up (y)	27	14.5
Sex	Male	Female
Eye movement abnormalities	Nystagmus, oculomotor apraxia	Nystagmus, oculomotor apraxia
Bulbar abnormalities	Dysarthria	Dysarthria
Upper motor neuron	Spastic paraparesis, pyramidal signs	Spastic paraparesis, pyramidal signs
Lower motor neuron	Absent ankle jerk, sensory loss, and wasting of small foot muscles	Absent ankle jerk, sensory loss, and wasting of small foot muscles
Movement disorders	Limb ataxia, dystonia of upper limbs and neck	Limb ataxia, chorea
Age at wheelchair dependence (y)	15	10
Epileptic seizures	Focal seizures	None
Cognitive impairment	Moderate to severe	Moderate to severe
Telangiectasia	Conjunctival	Conjunctival
Skin changes	Few vitiligo-like patches	Prominent vitiligo-like
Alpha-fetoprotein (AFP)	521 ng/ml at age 22 (strongly elevated)	457 ng/ml at age 10 (strongly elevated)
Brain MRI	Cerebellar atrophy	Cerebellar atrophy

### Genetic evaluation

We performed exome sequencing in both living patients (II:1, II:4, Fig. 1). Only the two heterozygous variants in the *ATM* gene shown in Table 2 withstood the filtering process as described in materials and methods. Variant 1 is a one base pair deletion leading to an immediate truncation of the large (3056 aa) ATM serine/threonine kinase after amino acid 128. This variant almost certainly results in a loss of function of the ATM protein and has previously been reported as pathogenic (Table 2) [11–13]. Variant 2 is a missense variant that has a CADD score of 23.1 which is nearly identical to variant 1 (23.3) but is classified as benign by PolyPhen-2 and deleterious by MutationTaster (Table 2). Analysis of the data deposited in ClinVar shows that AT-causing variants are relatively evenly distributed over the whole ATM protein, including the region which contains variant 2 (data not shown). Variant 2 has previously been reported as variant of uncertain significance (VUS) or benign because it has also been observed in healthy controls (Table 2) [14]. However, an allele frequency of 1.03e−04 in gnomAD does not argue against causality for a recessive disease. In addition, Coutelier et al. found this variant in the homozygous state in one patient and reported it to be most likely causative [15]. Both variants segregate with disease and are located in trans in the examined family. They are the only variants in *ATM* and genes causative for related phenotypes which were identified in our exome data using a fairly relaxed filtering approach (CADD score > 15 and MAF < 1% and affecting protein sequence or splice sites). Therefore, our data suggest that both variants together cause AT in this family and that variant 2 should be regarded as causative.

**Table 2:** Variants in the *ATM* gene (GRCh38/hg38)

Genetic finding	Variant #1	Variant #2
Chromosome level	chr11.hg38:g.108235719del	chr11.hg38:g.108301655G>C
Genomic level	NC_000011.10:g.108235719del	NC_000011.10:g.108301655G>C
Coding sequence level	NM_000051.4:c.381del	NM_000051.4:c.5185G>C
Protein level	NM_000051.4(ATM_i001): p.(Val128*)	NM_000051.4(ATM_i001): p.(Val1729Leu)
CADD (Phred-scaled)	23.3	23.1
MutationTaster (score/class)	Not applicable	0.972/deleterious
PolyPhen-2 HVAR (score/class)	Not applicable	0.311/benign
gnomAD (MAF, nr. of alleles analyzed)	3.98e−06 (1/251238)	1.03e−04 (29/282530)
dbSNP (153 all)	rs587781831	rs3092907
HGMD (public 01.08.21)	Not listed	not listed
ClinVar	Pathogenic	VUS
LOVD	VUS, pathogenic	VUS

*CADD* combined annotation-dependent depletion, *dbSNP* database of single nucleotide polymorphism, *gnomAD* genome Aggregation Database, *HGMD* Human Gene Mutation Database, *MAF* minor allele frequency, *VUS* variant of uncertain significance

## Discussion

We show co-segregation and trans-positioning of two putatively causative *ATM* variants in an Egyptian family with AT. The clinical findings, elevated AFP, and inheritance pattern are typical for AT [2]. Elevated AFP serum levels are also found in ataxia with ocular apraxia types 2 and 4 (AOA2, AOA4). These are differential diagnoses of AT caused by variants in the senataxin (*SETX*) and the polynucleotide kinase 3′-phosphatase (*PNKP*) genes [16]. However, patients with AOA2/4 do not exhibit telangiectasia, and our patients did neither carry putatively causative *SETX* nor *PNKP* variants. While variant 1 (NM_000051.4(ATM_i001):p.(Val128*)) has already been regarded as causative, variant 2 (NM_000051.4(ATM_i001):p.(Val1729Leu)) has been classified as VUS or benign. Our data argue that this variant 2 is causative. Segregation, position in trans, low MAF in gnomAD, high CADD score, and the fact that we found no other putatively causative variants for AT or genes implied in the differential diagnoses of AT all point to a pathogenicity of the variant 2. Additionally, this variant in the homozygous state has previously been reported to be causative in a single AT patient [15]. In summary, we conclude that variant 2 is causative. Limitations of our study are the relatively small size of the family and a lack of functional data.

## Data Availability

Required data are stored and available upon request.
